# Dupilumab (Dupixent^®^) tends to be an effective therapy for uncontrolled severe chronic rhinosinusitis with nasal polyps: real data of a single-centered, retrospective single-arm longitudinal study from a university hospital in Germany

**DOI:** 10.1007/s00405-022-07679-y

**Published:** 2022-10-15

**Authors:** Florian Jansen, Benjamin Becker, Jördis K. Eden, Philippe C. Breda, Amra Hot, Tim Oqueka, Christian S. Betz, Anna S. Hoffmann

**Affiliations:** 1 Department of Otorhinolaryngology, University Medical Center Hamburg-Eppendorf, Martinistraße 52, 20246 Hamburg, Germany; 2 Institute of Medical Biometry and Epidemiology, University Medical Center Hamburg-Eppendorf, Martinistraße 52, 20246 Hamburg, Germany; 3 II. Medical Clinic and Polyclinic, Department of Pneumology, University Medical Center Hamburg-Eppendorf, Martinistraße 52, 20246 Hamburg, Germany; 4 Department of Otorhinolaryngology, Head and Neurocenter, University Medical Center Hamburg-Eppendorf, Martinistrasse 52, 20251 Hamburg, Germany

**Keywords:** CRSwNP, Dupilumab, Biologic, Monoclonal antibody, Nasal polyposis, Type 2 inflammation

## Abstract

**Introduction:**

Chronic rhinosinusitis with nasal polyps (CRSwNP) is an inflammatory disease, which is usually type 2-mediated in the western hemisphere, associated with severe therapeutic and socioeconomic challenges. The first targeted systemic treatment option for severe uncontrolled CRSwNP is a human monoclonal antibody against the interleukin-4 receptor α (IL-4Rα) subunit called dupilumab, which was approved for subcutaneous administration in Germany in October 2019. The purpose of this study is to investigate the efficacy of dupilumab in real life in patients treated with dupilumab in label according to license in our department in 2019–2021.

**Materials and methods:**

Since October 2019, we have investigated 40 patients (18 men, 22 women) treated with dupilumab in a single-center, retrospective single-arm longitudinal study. The following parameters were collected before treatment (baseline), at 1 month, 4 months, 7 months, 10 months, and 13 months: the Sino-Nasal Outcome Test-22 (SNOT-22), the forced expiratory pressure in 1 s (FEV-1), the olfactometry using Sniffin' Sticks-12 identification test (SSIT), a visual analog scale of the total complaints, the Nasal Polyp Score (NPS), histologic findings as well as total serum IgE, eosinophilic cationic protein in serum and blood eosinophils.

**Results:**

The average age was 52.7 years (± 15.3). The follow-up period was 13 months. The SNOT-22 average was 60 points (± 22.2) at the first visit, 28.2 points (± 17.1) after 4 months and 20.8 points (± 17.7) after 13 months. The NPS was 4.3 points (± 1.5), after 4 months 2.1 points (± 1.3) and after 13 months 1.4 points (± 1.1). Olfactometry showed 3.2 points (± 3.7) at the baseline, 7.0 points (± 4.0) after 4 months and 7.8 points (± 3.5) after 13 months. The other parameters also improved. Most parameters showed linear dependence in the slopes under therapy (*p* < 0.001). Adverse side effects were mostly only mild, and no rescue therapy was needed.

**Conclusion:**

There is a clear improvement in the medical condition and symptoms in all categories mentioned under therapy with dupilumab, as well as a reduction in the need for systemic glucocorticoids and revision surgery as rescue treatment. Our results show that dupilumab tends to be an effective therapy alternative for severe CRSwNP.

## Introduction

Chronic rhinosinusitis (CRS) is a collective term for inflammation of the nasal cavities and paranasal sinuses that is associated with severe clinical, therapeutic and socioeconomic challenges [1]. CRS is typically defined by nasal obstruction, excessive rhinorrhea with or without loss of olfactory function up to the point of anosmia or facial pressure pain. As a temporal cutoff, symptoms must be present for the total duration of 12 weeks or longer. Epidemiological the prevalence of CRS in Europe is considered at 13% of the overall population [2–4].

Furthermore, CRS can be divided into CRS with (CRSwNP) or without nasal polyps (CRSsNP) depending on phenotype, which is crucial for possible treatment options and prognosis of the disease course [2, 5]. An estimated 48% of CRSwNP patients have comorbid bronchial asthma, which is thought to negatively influence disease. To this end, studies showed that patients with comorbid bronchial asthma had significantly greater sinus inflammation than patients without bronchial asthma, as well as an increased likelihood of experiencing sinus surgery when severe lung disease is present [2, 6, 7]. In particular, there appears to be an association between the occurrence of CRSwNP and the need for sinus surgery when late-onset asthma (onset of disease after 18 years of age) with eosinophilic inflammation and a T-helper cell type 2 (Th2) response is present (type 2 asthma) [8–10]. In CRSwNP, a subtype entity referred to as non-steroidal anti-inflammatory drugs exacerbated respiratory disease (N-ERD) can be described [4]. This clinical pattern is characterized by the fact that the upper and lower respiratory tract are particularly sensitive to NSAIDS and can modulate the course of the disease to the negative [11]. In this regard, CRSwNP associated with N-ERD has been shown to lead to rapid recurrence of polyps after surgery, making salvage sinus surgery more difficult to perform as well as predicting a more severe course of N-ERD [12]. Current treatment options for CRSwNP mostly include systemic steroids and repeated sinus surgery. Even though treatment options are improving due to increasingly better, more precise surgical techniques (e.g., endoscopic paranasal sinus surgery), sufficient therapeutic success is not always possible. For these patients, there is a need for new treatment options [13–15].

Initial target strategies include the monoclonal antibody dupilumab (Dupixent^®^, Sanofi, Paris, France and Regeneron, NY, USA), that is approved for the treatment as subcutaneous administration of CRSwNP through the pivotal studies LIBERTY NP SINUS (LNPS)-24 and LNPS-52, which has been shown to potently suppress key inflammatory pathways in CRSwNP by inhibiting interleukin-4 (IL-4) and interleukin-13 (IL-13) signaling [16]. Clinical trials with real-life data from hospitals providing direct patient care are urgently needed to now test the transferability into clinical practice as well as the effectiveness of the drug. To meet this need, we are evaluating in this study the potential clinical efficacy of blocking this axis in CRSwNP by the biological dupilumab in adult patients treated at our university medical center in Hamburg–Eppendorf, Germany, between December 2019 and April 2021. In doing so, we strictly followed the indication criteria for biologic therapy based on the EPOS2020 criteria. These state that at least three criteria of the following are required: first, tissue eosinophilia of ≥ 10/high power field, or blood eosinophilia of ≥ 250 per microliter, or total serum IgE ≥ 100 IU/ml. Second, ≥ 2 courses of systemic corticosteroids per year, or long-term treatment with low-dose systemic corticosteroids (> 3 months). Third, an SNOT-22-Score of > 40 points. Fourth, anosmia on the smell test (score depending on the test). Fifth, asthma requiring regular inhaled corticosteroids. [17]

## Dupilumab and its potential effects on the pathomechanism in CRSwNP

Dupilumab is a fully human immunoglobulin G4 subclass monoclonal antibody that blocks IL-4 and IL-13 signaling by specifically binding to the IL-4Rα receptor subunit. Thus, it modulates cell function, cell signaling through several chemokines and immunoglobulin E (IgE) synthesis and should adjust the inflammation progress [18]. After subcutaneous administration, the bioavailability of dupilumab should be around 64% [19].

There are various explanations that could explain the better disease control by dupilumab and its effect on the pathomechanism leading to CRSwNP. One possible explanation is that dupilumab, when it binds primarily to IL-4Rα, inhibits IL-4 signaling and subsequent inflammation-promoting responses in this pathway. Consequently, by suppressing Th2 differentiation, it would downregulate essential onward steps to T2 inflammation, such as mast cell activity and IgE synthesis. In addition, auto-inflammatory cascades leading to eosinophilia are inhibited. However, if dupilumab is more likely to prevent IL-4Rα from associating with the IL-13Rα1 subunit, consequently, inhibition of IL-13-driven disease will predominate. Moreover, dupilumab may thus block both signaling pathways, IL-4 and IL-13, and thus modulate the inflammatory response and eosinophilia via both signaling pathways [4, 20]. Moreover, to show unique functions of the interleukin 4 receptors in vivo, studies in mouse models showed that for IL-4Rα and interleukin-13Rα1 (IL-13Rα1), interleukin-4-Rα/γC regulates Th2 cell responses, whereas the IL4-Rα/IL-13Rα1 type II receptor is particularly important for the induction of allergen-induced hypersensitivity in the lower airways and is thus crucial for the pathogenesis of type 2 asthma, suggesting that CRSwNP and type 2 asthma share some parts of the same pathogenesis [4, 21]. Based on these findings, it can be concluded that type 2 asthma and CRSwNP can both be affected by dupilumab, and thus studies are urgently needed to investigate this in real-life data on CRSwNP.

## Materials and methods

### Study design

The present study is a mono-center, retrospective single-arm longitudinal study. The primary aim of this study is to investigate the influence of dupilumab in real life on the long-term course on nasal symptoms (such as nasal obstruction, sinonasal symptoms, rhinorrhea, facial pain, and sleep disturbances), olfactory function, nasal polyps, pulmonary function, and blood counts (total serum IgE, eosinophil cationic protein (ECP), absolute eosinophil count) in patients with CRSwNP.

### Patients

40 adult patients (> 18 years) were included in this study at the outpatient center of the University Hospital Hamburg–Eppendorf from November 2019 until April 2021. The study was approved by the ethics committee of Hamburg, Germany (2020-10264-BO-ff).

### Inclusion criteria

Patients with severe uncontrolled chronic rhinosinusitis with nasal polyps (CRSwNP). This defines patients that got at least one nasal sinus surgery without a long-term benefit of their symptoms in their medical history, the continuous usage of topic corticosteroids in the maximum dosage and nasal rinses with saline, the therapy with systemic corticosteroids once a year or contraindications thereto. In addition, there should be evidence of a Th2-Inflammatory reaction in histopathological findings in nasal mucosal tissue of previous operations (more than 10 eosinophils per High-Power-Field), more than 100 kU/L of total serum IgE or more than 250 eosinophils per µl in blood samples. Moreover, these patients must suffer from anosmia and have a high symptom burden of an SNOT-22-Score over 40 Points. When patients met three or more criteria as described above, referring to the EPOS2020 criteria, the indication of a therapy with 300 mg of dupilumab, administered subcutaneously, every 2 weeks, was given, and were included in the retrospective analysis [2].

### Exclusion criteria

Patients with immunosuppressive diseases, cystic fibrosis, pregnancy or breastfeeding and patients under therapy with other biologics.

### Clinical findings and outcome measures

Age, gender, clinical history, the number of nasal sinus surgeries, the FEV-1 and the olfactometry were evaluated. Sniffin' Sticks-12 identification test was used in olfactometry [22]. In each case, the worse side was included in the analysis. Evaluation of visual analog scales (VAS) of the CRS-symptoms such as nasal obstruction, sinonasal symptoms, rhinorrhea, facial pain and sleep disorders and the SNOT-22 questionnaire were performed. Furthermore, the NPS was assessed by nasal endoscopy (score 1: small polyps in the middle meatus not reaching below the inferior border of the middle turbinate; score 2: polyps reaching below the lower border of the middle turbinate; score 3: large polyps reaching the lower border of the inferior turbinate or polyps medial the middle turbinate; score 4: large polyps causing complete obstruction of the inferior nasal cavity; sum of both nasal sides) [23]. Histologic findings (Eosinophils/high power field) as well as total serum IgE, ECP in serum and total eosinophils in whole blood were also recorded.

The coprimary endpoints were the NPS and VAS (nasal obstruction, sinonasal symptoms, rhinorrhea, facial pain and sleep disorders). FEV-1, olfactometry, SNOT-22 questionnaire and blood results (total serum IgE, ECP, and blood eosinophils) were set as secondary endpoints. All these primary and secondary endpoints were examined at baseline as well as in follow-up visits with respect to a linear trend of outcomes over time.

### Baseline and follow-up visits

Baseline characteristics were collected before therapy and subsequent examinations were performed at 1 month, 4, 7, 10, and 13 months in therapy. At every visit the primary and secondary endpoints were noted as described above.

#### Hypothesis

We assume that the mentioned parameters will improve in favor of sinusitic complaints in the sense of a reduction of the points in the questionnaires and visual analog scales, a reduction of the points in NPS, as well as an improvement of the points of olfactometry and FEV-1. Furthermore, we think that only a transient increase in the absolute eosinophil count in whole blood, the total serum IgE as well as the ECP in serum will be seen.

### Statistics

Categorical data are summarized by absolute and relative frequencies. Continuous data are summarized by mean, standard deviation, median, interquartile range, minimum, and maximum. These measures are presented for the total sample.

To model the linear trend of outcomes over time, mixed linear models were calculated with time as the fixed effect and patientID as the random intercept. The target variable is the respective outcome under consideration. The baseline variable was included in the model as a covariate. If a linear trend is assumed over time and across all patients, then the slope (+ 95% CI) indicates how the respective target variable changed on average every 3 months.

## Results

### Characterization of patients

40 patients (18 male, 22 female) were included in our study. The average age was 52.7 years (SD ± 15.3) with a minimum age of 20 and maximum age of 84 years. The follow-up period was 13 months. 57% of the patients had a history with confirmed allergies (including allergic rhinitis (seasonal or perennial), allergic conjunctivitis, allergic contact dermatitis, food or drug allergy in the patient's history or other allergic reactions documented by a treating physician) and 88% of the patients had a history of bronchial asthma. 21 patients had known N-ERD (defined as the presence of CRSwNP, an asthma requiring therapy as well as hypersensitivity reactions to NSAIDs in the patient's history). The average number of FESS before biological treatment was 3.7 (± 1.9). Out of 22 available histopathological reports all showed more than 10 eosinophils per high power field (see Table 1).

**Table 1:** Baseline characteristics of the cohort

Baseline characteristics	*N* = 40
Gender	
Male	18 40 (45%)
Female	22/40 (55%)
Age in years	
Mean (SD)	52.70 (15.34)
Median (IQR)	52 (43.75, 62.00)
Range	20.00, 84
Patients with allergies in percentage	23/40 (57%)
Patients with bronchial asthma in percentage	35/40 (88%)
Patients with NSAID-exacerbated respiratory disease in percentage	21/39 (54%)
Number of functional endoscopic sinus surgeries per patient in percentage	
Mean (SD)	3.72 (1.88)
Median (IQR)	3 (3.00, 4.00)
Range	1.00, 10
Histopathological findings of eosinophils per high power field in percentage	
> 10/high power field	4/22 (17%)
> 100/high power field	16/22 (70%)
> 200/high power field	2/22 (8.7%)
SinoNasal Outcome Test-22 in points	
Mean (SD)	60.48 (22.17)
Median (IQR)	60 (44.75, 79.50)
Range	14.00, 101
Nasal polyps score in points	
Mean (SD)	4.30 (1.47)
Median (IQR)	4 (4.00, 4.25)
Range	2.00, 8
Sniffin‘ Sticks-22 identification test in points	
Mean (SD)	3.22 (3.74)
Median (IQR)	1 (0.00, 6.00)
Range	0.00, 11
Visual analog scale of facial pain in points	
Mean (SD)	4.42 (3.59)
Median (IQR)	4 (0.75, 7.00)
Range	0.00, 10
Visual analog scale of nasal blockage in points	
Mean (SD)	6.05 (3.00)
Median (IQR)	7 (4.00, 8.00)
Range	0.00, 10
Visual analog scale of sleep disorder in points	
Mean (SD)	5.40 (3.53)
Median (IQR)	5 (2.00, 9.00)
Range	0.00, 10
Visual analog scale of rhinorrhoe in points	
Mean (SD)	6.70 (2.94)
Median (IQR)	7 (5.00, 9.25)
Range	0.00, 10
Forced expiratory pressure in 1 s in percentage points	
Mean (SD)	73.88 (15.40)
Median (IQR)	74 (66.50, 86.25)
Range	40.00, 10

### Nasal polyp score before and under treatment

**Fig. 1:**
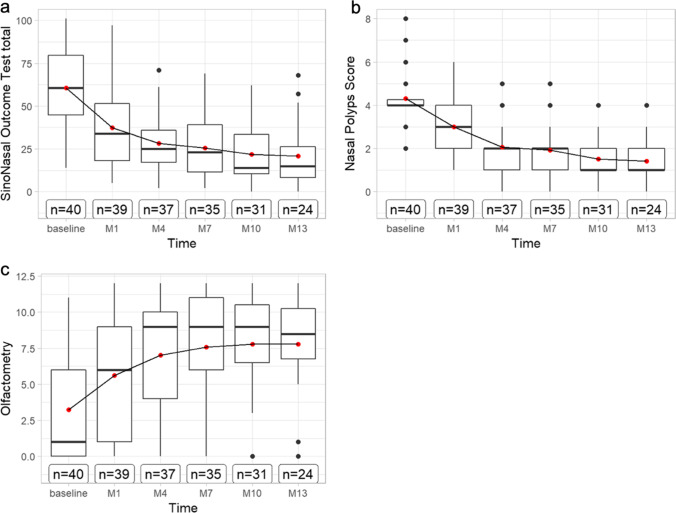
Boxplots displaying **a** SinoNasal Outcome Test-22 results, **b** change in Nasal Polyps Score and (c) olfactometry using Sniffin’ Sticks-12 identification test in points over baseline to 1 month, 4 months, 7 months, 10 months, and 13 months on dupilumab therapy. *M* months

The NPS was 3.0 points (± 1.4) after 1 month and improved after 4 months to 2.1 points (± 1.3), to 1.9 points (± 1.4) after 7 months, 1.5 points (± 1.1) after 10 months and up to 1.4 (± 1.1) at the end of this study, respectively (see Fig. 1) Here, the slopes show linear dependence (− 0.45 to − 0.29, *p* < 0.001) (see appendix Fig. 1).

### VAS-scores and SNOT-22 questionnaire before and under treatment

The average SNOT-22 questionnaire score before treatment was 60 points (± 22.2). The average score improved after 1 month to 37.3 points (± 22.9), after 4 months up to 28.2 (± 17.1), to 25.6 points (± 18.2) after 7 months, to 21.8 points (± 16.7) after 10 months and at the end of the study to 20.8 points (± 17.7)) (see Fig. 1). Here, the slopes show toward linear dependence (− 4.18 to 2.15, *p* < 0.001), too (see Appendix Fig. 6).

**Fig. 2:**
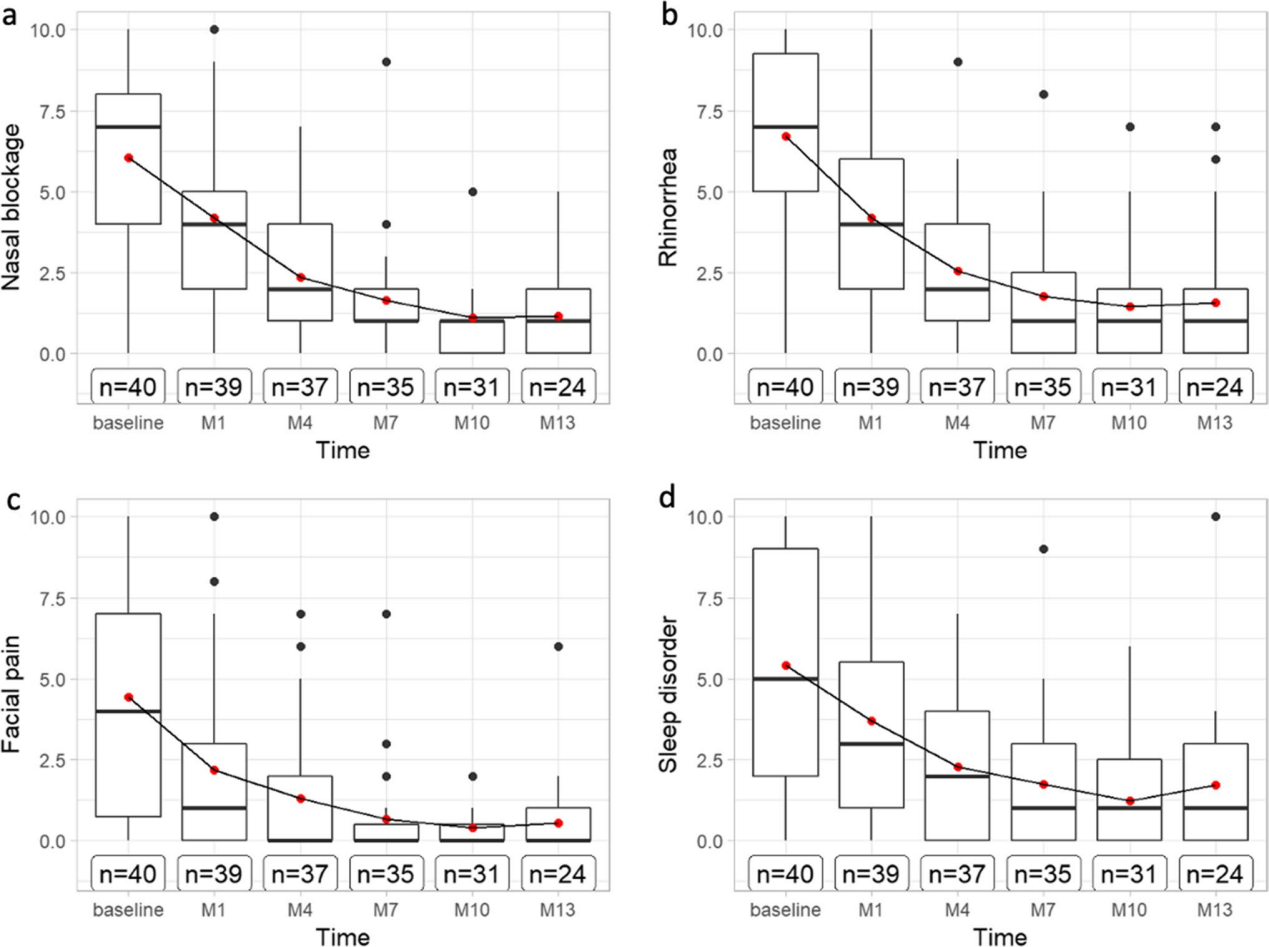
Boxplots displaying the results of the Visual Analog Scale of **a** nasal blockage, **b** rhinorrhea, **c** facial pain and **d** sleep disorder in points over baseline to 1 month, 4 months, 7 months, 10 months, and 13 months on dupilumab therapy. *M* months

The scores of the VAS on the baseline visits were ± 6.0 points (± 3.0) regarding nasal airway obstruction, 7.2 points (± 2.0)-related sinusitic complaints, 6.7 points (± 2.9) based on rhinorrhea, 4.4 points (± 3.6) regarding facial pain and 5.4 points (± 3.5) on sleep disorder. All VAS-scores improved markedly under therapy (see Fig. 2). Furthermore, all scores show a linear dependence in the slopes (see Appendix Figs. 7, 8, 9, 10 (− 0.94 to − 0.6, − 0.83 to − 047, − 0.55 to − 0.23, − 0.74 to − 038, all *p* < 0.001)).

### Olfactometry and FEV-1 before and under treatment

The olfactometry results were 3.2 points (± 3.7) at the baseline, 5.6 points (± 4.2) after 1 month, 7.0 points (± 4.0) after 4 months, 7.6 points (± 3.7) after 7 months, 7.8 points (± 3.6) after 10 months and 7.8 points (± 3.5) after 13 months. Furthermore, it improved clearly after 10 and 13 months (see Fig. 1). Thus, the smelling improved from an anosmia almost to a normosmia after 13 months with a linear dependence in the slopes (0.42 to 0.8, *p* < 0001).

**Fig. 3:**
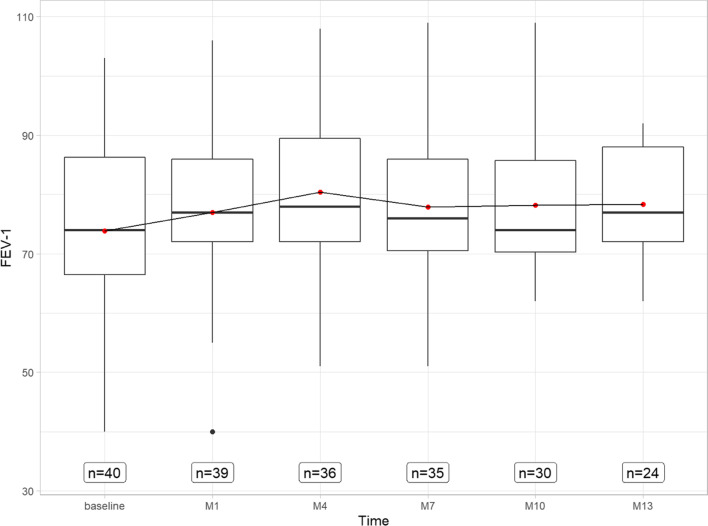
Boxplot displaying the results of the forced expiratory pressure in 1 s in percentage points over baseline to 1 month, 4 months, 7 months, 10 months, and 13 months on dupilumab therapy. FEV-1: forced expiratory pressure in 1 s; *M* months

The average FEV-1 was 73.9% (± 15.4) before treatment and slightly improved to 77.0% (± 12.9) after 1 month. From then, it was 80.4% (± 11.4) after 4 months, 77.9% (± 12.7) 7 months, 78.2% (± 11.1) after 10 months and 78.4 (± 9.8) after 13 months (see Fig. 3). Only a lower slope factor was found here ((see appendix Fig. 7 (− 0.09 to 1.13, *p* = 0.088).

### Laboratory chemical changes before and under treatment

**Fig. 4:**
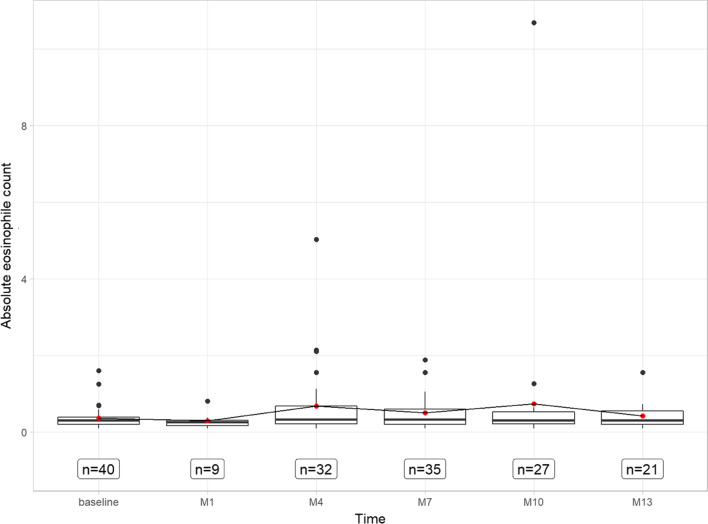
Boxplot displaying the progression of absolute eosinophile count in billions per liter over baseline to 1 month, 4 months, 7 months, 10 months, and 13 months on dupilumab therapy. *M* months

The average ECP was 35.3 µg/l (± 25.2) in serum, the total serum IgE 308.4 kU/l (± 554.9) and the absolute eosinophils 0.5 billion/l (± 0.3) in whole blood at the baseline visit. There were not any substantial changes at the ECP throughout the study period. The average absolute eosinophils changed to 0.3 billion/l (± 0.2) after 1 month and increased markedly to 0.7 billion/l (± 1.0) after 4 months, to 0.5 billion/l (± 0.5) after 7 months and to 0.7 (± 2.0) after 10 months and to 0.4 billion/l after 13 months (± 0.3) (see Fig. 4 and appendix Fig. 12). However, the total serum IgE decreased to 64.7 kU/l (± 94.2) at the end of the study. Here, no linear dependence was shown.

### Safety aspects and rescue therapies

The dupilumab therapy was well-tolerated. Two patients had mild joint pain and one had an obstipation tendency under therapy, three patients suffered from mild xerophthalmia and in one case a keratoconjunctivitis. Another three patients suffered from mild herpes labialis, which, however, was well-controlled with topical therapy. We documented a case of genital herpes, which spontaneously regressed. One patient had mild xeroderma in the midface, but this also recovered after a few weeks with local therapy.

In one patient we diagnosed moderate myalgias and cephalgias that caused the discontinuation of the therapy. In addition, we documented another patient who independently discontinued the medication for 8 weeks due to a vacation trip without consultation, but continued to show good disease control after continuation of treatment. After admission, the patient showed a blood eosinophilia of 1.89 billion per liter, so that a pulmonologist and a rheumatologist also evaluated the patient and decided to continue the therapy.

Last but not least, we have documented a case of severe angioedema of the right upper and lower eyelid after 4 months of therapy, but a direct link to therapy was unclear. Thus, we continued the therapy after allergological co-evaluation, and the angioedema did not reoccur.

We documented no non-responders under therapy. No rescue therapies in the form of systemic corticosteroids or revision surgery were necessary during the study. Furthermore, there were no infections in the sense of a possible immunosuppression.

## Discussion

For the first time, there is now the possibility to use an antibody for targeted therapy in a disease that is often difficult to treat specifically, away from systematic corticosteroids and recurrent paranasal sinus surgery. Now, a lot of data from clinical practice is needed to see if this treatment model can be implemented in clinical practice and if the double signal blockade of IL-4 and IL-13 can provide the desired success and disease control. In this study, we strictly adhered to the EPOS2020 criteria to see if we could see a regular benefit for our patients with uncontrolled CRSwNP in a clinical setting, reduce the need for surgery and cortisone therapy, and still provide a safe form of therapy. In this study on a limited number of patients and under the parameters above, we saw a clear improvement in the symptoms of CRSwNP and an improved lung function with only rare complications and no need for rescue therapy. On one hand, the coprimary endpoints in the form of the VAS and the NPS improved markedly, so that objective, as well as subjective improvement was shown. On the other hand, the SNOT-22 questionnaire and olfactory ability improved substantially as secondary endpoints, resulting in a better quality of life for the patients. A linear trend can be seen here over time and across all patients. This improvement of the objectively reduced nasal polyps and the subjective improvement of sinusitic complaints and nasal blockage caused by the steady reduction of nasal polyps fits the known registration studies [16]. The statistical evidence of a linear dependence over time, however, can be an interesting finding to help clinically active rhinologists in everyday life, on one hand to estimate the further course of the disease in case of an improvement and on the other hand to give the patients a well-founded prognosis in the context of the therapy. We, therefore, recommend that the VAS and the SNOT-22 questionnaire on one hand and the NPS and olfactometry on the other hand are firmly established as outcome markers in everyday clinical practice during therapy with dupilumab.

The strength of this study lies in its real context from a diverse cohort of patients from Hamburg, the second biggest city in Germany. It reports a cohort with standardized indication criteria, treatment regimen and follow-up schedule for 13 months. The therapeutic success as well as adverse events were monitored for 13 months, providing a benchmark for decisions in the therapy of CRSwNP for otolaryngologists.

The results of our study are consistent with the results of other studies [24]. However, we have seen in our cohort that the FEV-1 also improved during the course of therapy, leading to better control not only of sinusitic symptoms but also of improved lung function. This is also in line with other studies looking at both diseases under therapy with dupilumab, as they both can share a somewhat similar pathomechanism [25]. Epidemiologic and clinical studies clearly suggest that upper respiratory disease often accompanies lower respiratory disease and, therefore, type 2 asthma [26–28]. In patients with CRSwNP, type 2 asthma is the most common accompanying inflammatory disease. The frequency of co-occurrence increases again with the severity of the disease [6, 29, 30]. These patients suffer even more from the symptoms of CRSwNP, as both diseases increase the clinical burden with more severe nasal obstruction and olfactory loss, poorer lung function and asthma control and, and poorer health-related quality of life [31–33]. Considering these circumstances, it is even more important for clinicians to identify these patients and provide therapy that treats both conditions. Regarding this, the benefits of the therapy in monetary terms, especially the cost to the health care system, remain unclear and insufficient data make a direct comparison with other treatment methods difficult. If dupilumab can treat CRSwNP and severe asthma likewise such an approach may indeed prove cost-effectiveness and sustainability. The evaluation of the total costs of such treatment approaches should be taken into account in the future given that these are long-term and potentially life-long therapies. However, if the therapy can avoid repeated rescue therapies and potentially reduce medication and hospitalization rates, and if the prices of biologics are reduced by the approval of additional agents, this could reduce the cost to the healthcare system.

Furthermore, we saw a remarkable increase in absolute eosinophils in whole blood as a secondary endpoint under therapy with dupilumab, even though no linear trend could be seen here. Changes in blood eosinophils on dupilumab therapy varied by disease type, with minimal changes in atopic dermatitis, transient increases readily followed by decreases in asthma and CRSwNP, and significant decreases in eosinophilic esophagitis. The authors of the LIBERTY NP SINUS (LNPS)-24 and the LNPS-52-Trial postulate that the transient blood eosinophilia can be due to a decrease in eotaxin-3, which prevents eosinophils from migrating from the serum to the tissues. The reduction of total serum IgE has already been seen in other studies as well, since IL-4 is also required for immunoglobulin isotype switching to IgE, an important downstream mediator in the type 2 adaptive immune response, and dupilumab interferes in this cascade [16, 34–36]. The lack of response of ECP in blood as a secondary endpoint to dupilumab is only partially consistent with other studies. Here, tissue samples of nasal mucosa and nasal secretions showed that several type 2 biomarkers, including ECP, were downregulated [16, 37]. Although ECP levels were shown to be related to the number of activated eosinophils in the blood and these were also increased in patients with CRSwNP, no significant correlation and only transient increases in ECP in the blood were demonstrated during therapy with dupilumab [38–41]. The lack of a persistent response to ECP concentration in blood is clinically interesting with regard to the cytotoxic effect of ECP at elevated levels, as previous studies have shown a damaging effect on nasal mucosal epithelium, corneal epithelium, lung surfactant structure, and tracheal epithelium, smooth muscle cell impairment, and an association with tissue remodeling, demonstrating a link with respiratory disease and eosinophilic esophagitis [42–48]. In this context, it would be interesting in further studies whether a change of concentration of ECP is found in the nasal mucosa or secretions in our cohort. However, since no permanent response was found in the concentration of serum ECP and dupilumab therapy and we saw no relevant side effects occur, our cohort showed no recommendation to establish serum ECP as a fixed outcome marker.

Limitations also apply to this study. Since only purely descriptive values of a real world cohort are presented here, we decided to present the trend of improvement in terms of the slope factor of the change in the parameters of the patients under dupilumab. A dedicated statement about the effectiveness of the drug can, therefore, only be made in terms of tendencies. Selection bias may also have occurred, due to the fact that the recruiting hospital was the university medical center, so that perhaps patients with more severe symptoms were referred. Thus, a transfer of our results to the clinical routine of colleagues practicing purely in private practice may be difficult. As with any new medication, hypervigilance and monitoring of patients will be needed until further safety data are available. It is essential that the right agent is given to the right patient, and as more biologics that target key points of the T2 inflammatory cascade appear on the horizon, identification of disease traits and associated biomarkers that predict treatment response with a particular biologic will become even more important. This underscores the importance of regularly updated clinical guidelines (such as EPOS) which help—in addition to a detailed history and clinical examination, as well as histopathologic and blood testing when appropriate—to identify patients with a Th2 inflammatory response.

To treat these patients adequately, a multidisciplinary approach with ear, nose and throat surgeons working alongside with pulmonologists, dermatologists and rheumatologists must become the norm and not the exception. In particular, the joint work with pulmonary specialists is essential, since a change in lung function under therapy with dupilumab and a high comorbidity with type 2 asthma.

Regarding the safety of therapy, only minor adverse events were observed for the most part, and only in one case therapy was discontinued due to complaints that may have been associated with the therapy. In the pivotal studies LIBERTY NP SINUS-24 and LIBERTY NP SINUS-52, as well as other studies, mostly mild adverse events (such as headache, injection reactions, worsening of sinusitic or asthmatic symptoms) were found, similar to our cohort. In the pivotal trials, however, these adverse events were found more frequently in the placebo group. In comparison, more adverse events were found in our cohort (in 14 of 40 patients), but in the absence of a control group, it is questionable whether there is always a direct relationship to drug administration. In general, a safe adverse event profile was shown [16, 37, 49, 50].

## Conclusions

Our study with real life data from a university medical center confirms the statement that dupilumab tends to be an effective therapy alternative for severe, uncontrolled CRSwNP. Under dupilumab therapy, there was a remarkable improvement in the medical condition and symptoms in all categories investigated, as well as the absence of a need for systemic glucocorticoids or rescue revision surgery was necessary with mostly only mild adverse side effects.

## Figures and Tables

**Fig. 5:**
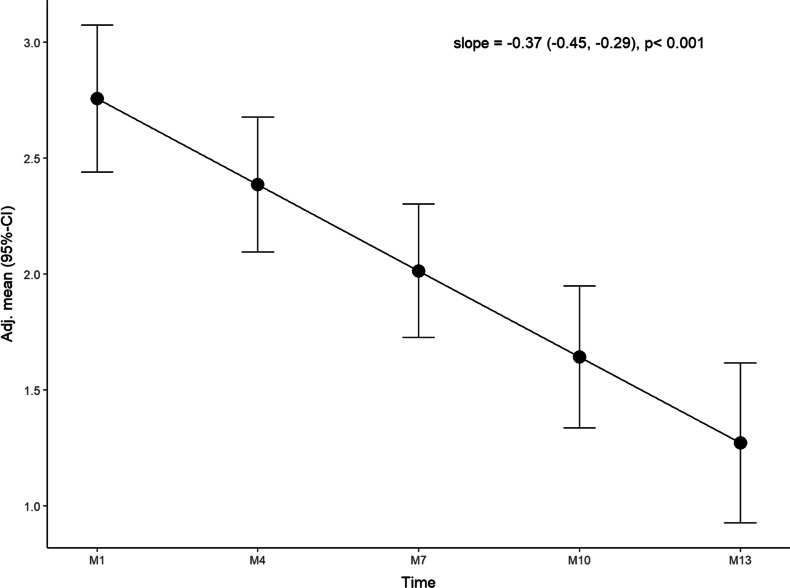
Slope displaying the linear dependence of the change of Nasal Polyps Score over 1 months, 4 months, 7 months, 10 months, and 13 months on dupilumab therapy. *M* months

**Fig. 6:**
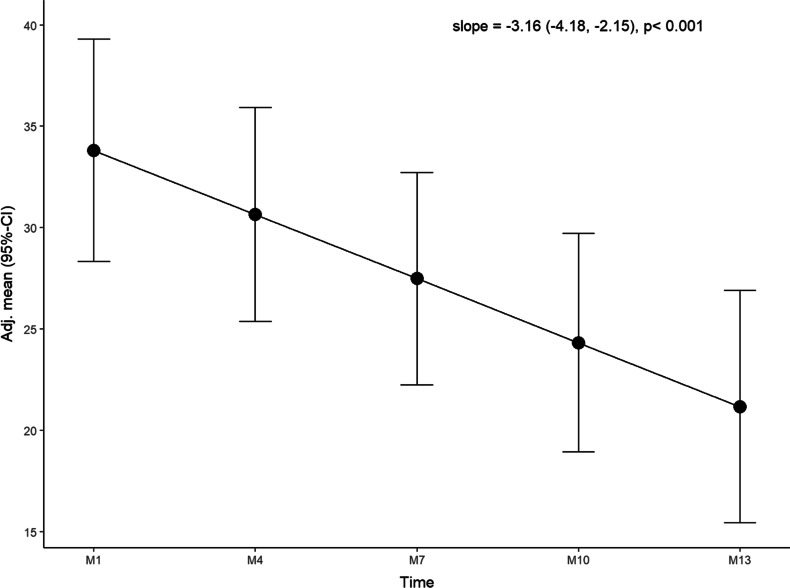
Slope displaying the linear dependence of the change of the SinoNasal Outcome Test-22 results over 1 months, 4 months, 7 months, 10 months, and 13 months on dupilumab therapy. *M* months

**Fig. 7:**
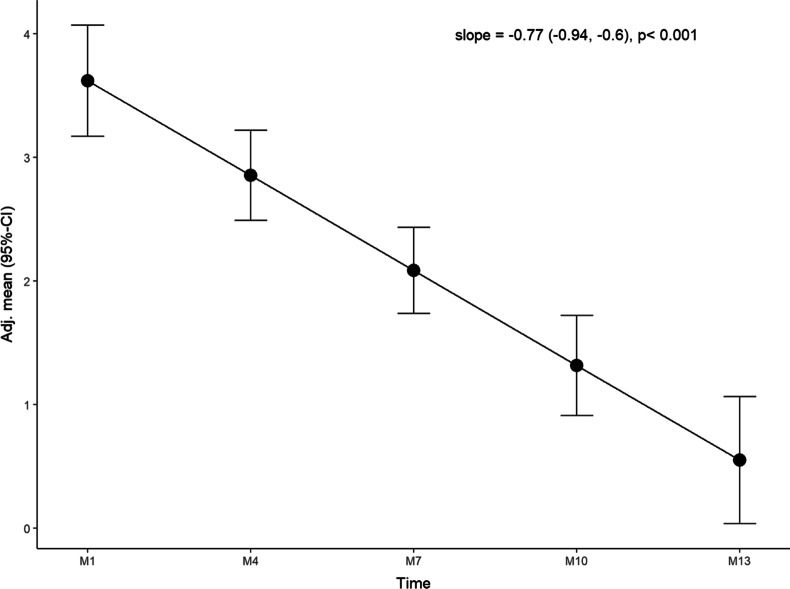
Slope displaying the linear dependence of the change of the points of the visual analog scale regarding nasal airway obstruction over 1 months, 4 months, 7 months, 10 months, and 13 months on dupilumab therapy. *M* months

**Fig. 8:**
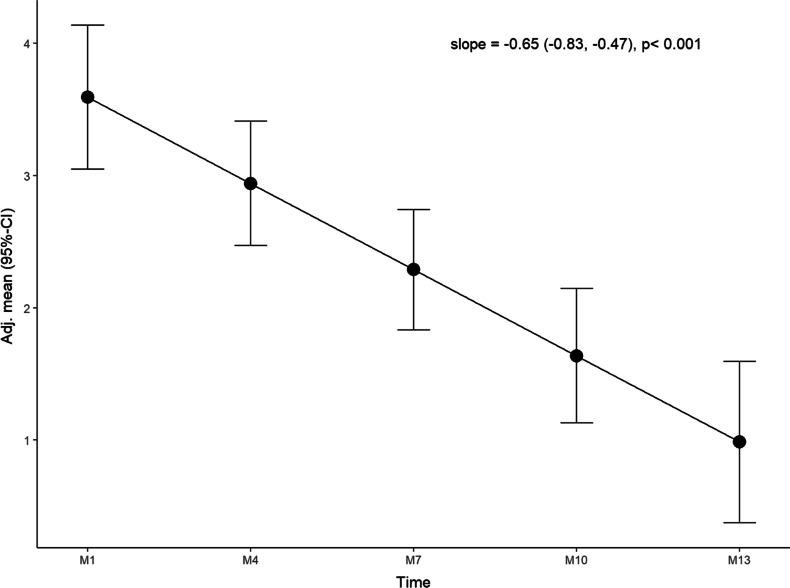
Slope displaying the linear dependence of the change of the points of the visual analog scale regarding rhinorrhea over 1 months, 4 months, 7 months, 10 months, and 13 months on dupilumab therapy. *M* months

**Fig. 9:**
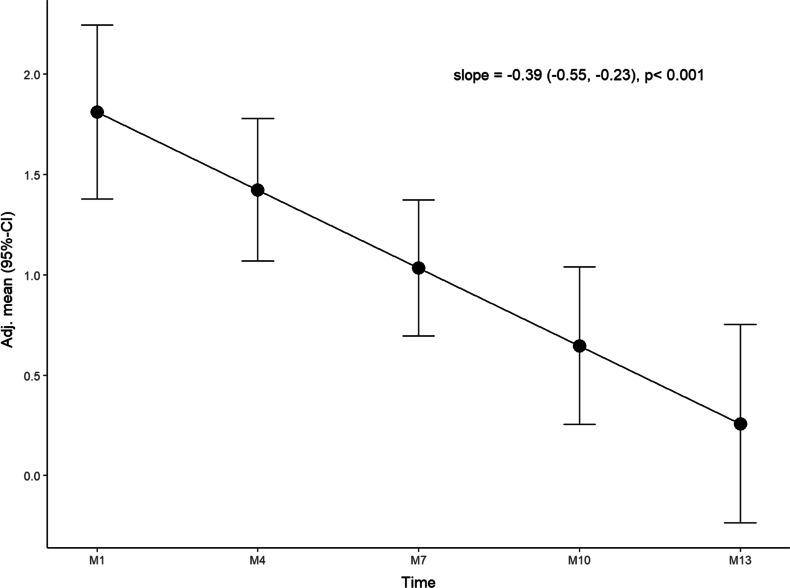
Slope displaying the linear dependence of the change of the points of the visual analog scale regarding facial pain over 1 months, 4 months, 7 months, 10 months, and 13 months on dupilumab therapy. *M* months

**Fig. 10:**
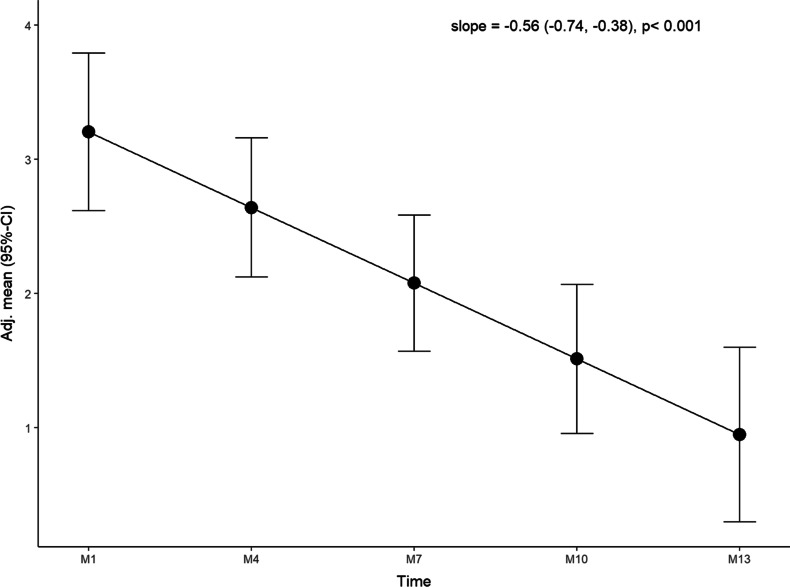
Slope displaying the linear dependence of the change of the points of the visual analog scale regarding sleep disorder over 1 months, 4 months, 7 months, 10 months, and 13 months on dupilumab therapy. *M* months

**Fig. 11:**
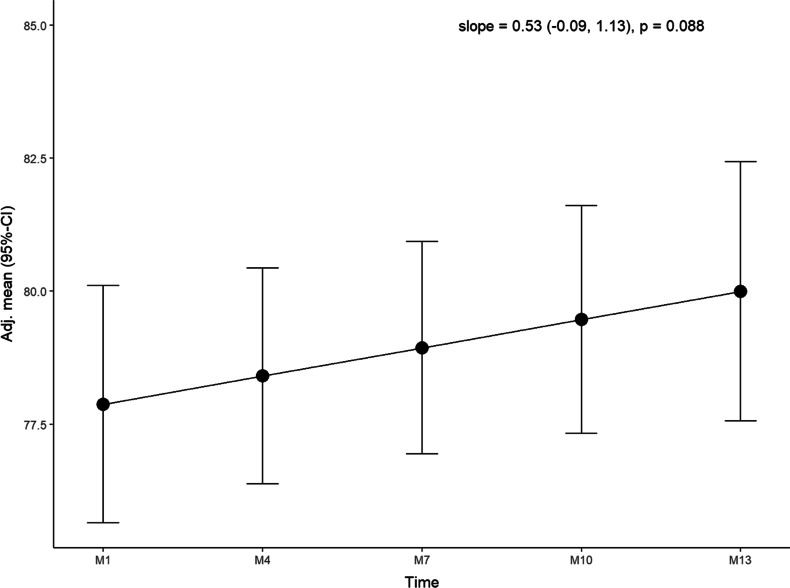
Slope displaying the linear dependence of the change in percentage of the forced expiratory pressure in 1 s over 1 months, 4 months, 7 months, 10 months, and 13 months on dupilumab therapy. *M* months

**Fig. 12:**
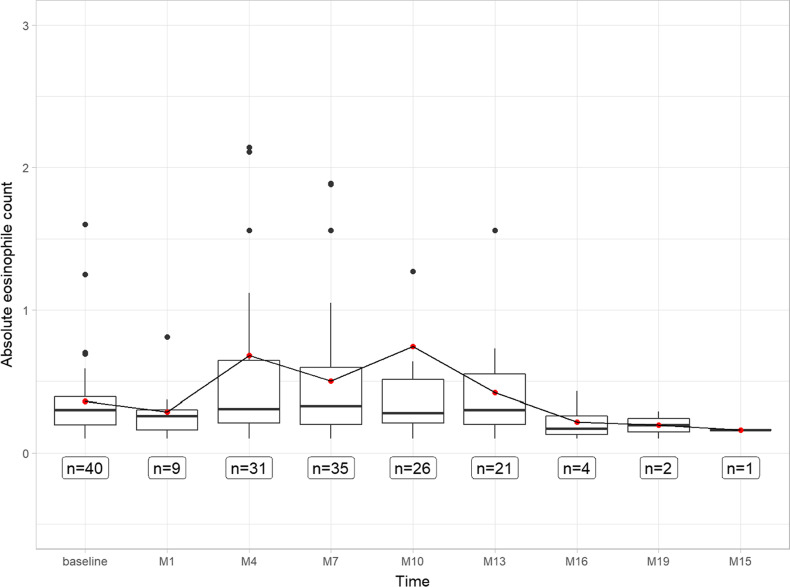
Boxplot displaying the progression of absolute eosinophile count in billions per liter over baseline to 1 month, 4 months, 7 months, 10 months, and 13 months on dupilumab therapy. For better presentation, the data of two individuals are not shown at time M4 and M10, respectively. *M* months

## Appendix

See Figs. 5, 6, 7, 8, 9, 10, 11 and 12.
